# A Machine Learning-Based Study of the Effects of Air Pollution and Weather in Respiratory Disease Patients Visiting Emergency Departments

**DOI:** 10.1155/2022/4462018

**Published:** 2022-02-02

**Authors:** Eu Sun Lee, Jung-Youn Kim, Young-Hoon Yoon, Seoung Bum Kim, Hyungu Kahng, Jinhyeok Park, Jaehoon Kim, Minjae Lee, Haeun Hwang, Sung Joon Park

**Affiliations:** ^1^Department of Emergency Medicine, Korea University College of Medicine, Seoul, Republic of Korea; ^2^School of Industrial Management Engineering, Korea University, Seoul, Republic of Korea

## Abstract

**Background:**

To date, investigating respiratory disease patients visiting the emergency departments related with fined dust is limited. This study aimed to analyze the effects of two variable-weather and air pollution on respiratory disease patients who visited emergency departments.

**Methods:**

This study utilized the National Emergency Department Information System (NEDIS) database. The meteorological data were obtained from the National Climate Data Service. Each weather factor reflected the accumulated data of 4 days: a patient's visit day and 3 days before the visit day. We utilized the RandomForestRegressor of scikit-learn for data analysis.

**Result:**

The study included 525,579 participants. This study found that multiple variables of weather and air pollution influenced the respiratory diseases of patients who visited emergency departments. Most of the respiratory disease patients had acute upper respiratory infections [J00–J06], influenza [J09–J11], and pneumonia [J12–J18], on which PM_10_ following temperature and steam pressure was the most influential. As the top three leading causes of admission to the emergency department, pneumonia [J12–J18], acute upper respiratory infections [J00–J06], and chronic lower respiratory diseases [J40–J47] were highly influenced by PM_10_.

**Conclusion:**

Most of the respiratory patients visiting EDs were diagnosed with acute upper respiratory infections, influenza, and pneumonia. Following temperature, steam pressure and PM_10_ had influential relations with these diseases. It is expected that the number of respiratory disease patients visiting the emergency departments will increase by day 3 when the steam pressure and temperature values are low, and the variables of air pollution are high. The number of respiratory disease patients visiting the emergency departments will increase by day 3 when the steam pressure and temperature values are low, and the variables of air pollution are high.

## 1. Introduction

Because of the exacerbation of air pollution, interest in the health effects of fine dust has increased. Fine dust is well known as a group 1 carcinogen. In addition, there have been reports of fine dust-related deaths, paralysis, neuropathy, high blood pressure, cardiovascular, and respiratory diseases [1–5] According to recent studies, it causes depression and anxiety [1], neurodegenerative diseases including dementia or Parkinson's disease, and skin diseases and increases the risk of childhood disorders, such as autism spectrum disorder, developmental disorders [6], asthma, respiratory tract infections, and atopic dermatitis [2, 7–9]. Nevertheless, there is no relevant research investigating patients visiting the emergency departments (EDs).

Overcrowding in EDs is a global problem and has been addressed as a national crisis in some countries [10]. The medical resources needed in the ED vary according to the severity, type of visit, and the patient's disease. Forecasting emergency medical demand can be a good way to efficiently allocate limited resources [11]. A variety of studies have evaluated the factors influencing the demands for emergency medical service [12]. In particular, previous studies have reported the characteristics of patients visiting EDs and the number of patients according to seasons and weather conditions [13].

Some diseases are sensitive to climate change. Studies have been conducted on the characteristics and number of patients visiting the ED depending on the season and climate. In addition, numerous studies have revealed that weather and air pollution are closely correlated with the development of cardiovascular and respiratory diseases. However, there is a lack of research on the multivariate factors in existing studies. Studies on the impact of weather and air pollution both on the demand for respiratory emergency medical resources remain insufficient.

Therefore, the data of respiratory disease patients who visited EDs were extracted from the national database of EDs and, using a machine learning technique, analyzed for the complex effect of air pollution, weather, and characteristics of respiratory disease patients visiting the ED for 3 years. Based on the analyzed general characteristics (age, gender, diagnosis), the use day of ED and hospital resources was examined. This study will help provide fundamental data on the prediction model of emergency respiratory patient visits related to weather including air pollution for patient treatment and the efficient management of limited medical resources.

## 2. Materials and Method

This study utilized the National Emergency Department Information System (NEDIS) database; a secondary data analysis was conducted using random forest (RF), a machine learning technique. NEDIS, an ED information network operated by the Ministry of Health and Welfare, is managed by the National Emergency Medical Center [14]. Since the execution of the system in 2003, it has collected clinical and administrative data of all patients who visited EDs nationwide. Korea provides national medical insurance, which covers 98% of the Korean population [15]. Therefore, the data collected are extremely influential. Emergency medical centers in the country undergo evaluation once a year in order to be approved as official organizations and automatically transmit all the digitalized data for the items requested by the NEDIS, as a principle. Therefore, the data utilized in this study included all the data from the EDs in Seoul, Korea.

## 3. Study Design and Statistical Analysis

Each weather factor reflected the accumulated data of 4 days: a patient's visit day and 3 days before the visit day. The number of explanatory variables corresponding to the response variable Y is 48 (4 × 12). With the use of weather and air pollution variables (*X*) such as temperature, the amount of precipitation, and PM_2,5_, the number (Y) of the ED patients who had a particular disease code was estimated. A RF Regression model that can select important variables was applied. The importance of an explanatory variable that influences a dependent variable was extracted via calculating impurity-based feature importance. We used the code available in the RandomForestRegressor of scikit-learn package. Pandas package (version 1.0.0; NumFOCUS, Austin, TX, USA) and Dask package were used mainly for data preprocessing.

**Figure 1:**
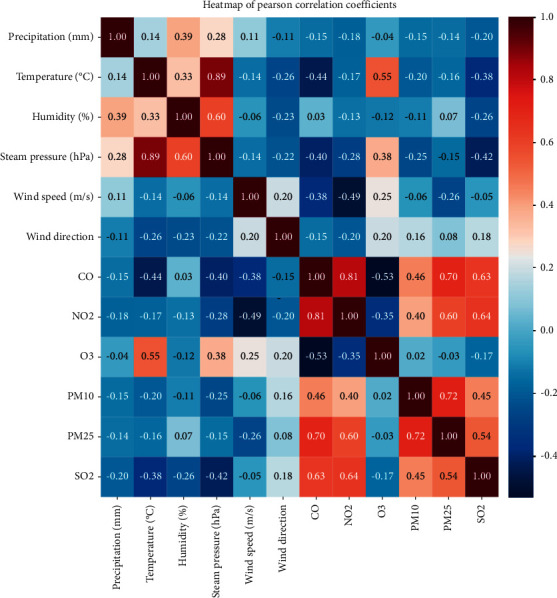
Heatmap showing the relations among the 27 predictor variables.

RF, as a machine supervised learning technique, has a combined form of multiple decision trees. In a conventional decision tree technique, if the number of explanatory variables is large, the number of the branches in one decision tree is also large. As a result, overfitting (in which the learned data only fits well) occurs. To prevent such overfitting, the RF randomly samples a part of the explanatory variables when one decision tree is generated and thereby creates multiple decision trees by sampling with replacement. Among the values predicted by the multiple decision trees generated in the process, the most predicted value becomes the final prediction value. In this study, the number of explanatory variables is large, and multicollinearity exists (Figure 1). For this reason, RF was applied rather than a conventional decision tree technique. To evaluate the performance of RF, Out of Bag, which evaluates performance with 1/3 of the data not used at the time of sampling with replacement, was used. The importance of an explanatory variable that influences a dependent variable was extracted. The most predictive features of regressors build up on models showing R ^∧^ 2 over 0.5.

## 4. ER Visit Data

Among the patients who had visited emergency medical centers in Seoul within the 36-month period from January 1, 2015, to December 31, 2017, those whose disease classification code (J code; J00–J99) at the time they left the ED was related to respiratory diseases according to the Korean Standard Classification of Diseases (KCD) (based on ICD-10) were selected. The analysis was performed using the first primary diagnosis in the emergency centers. Local emergency medical centers that failed to transmit KTAS were excluded from the analysis. The patients whose visit date and time were not recorded were excluded as well. The age, gender, disease name, and date and time of visit of study patients were utilized. The names of diseases are provided in Appendix 1.

## 5. Air Pollution and Weather Data

Fine dust contains enormous kinds of air pollutants, including heavy metals, ions, organic carbons, and black carbons. According to particle size, a particulate matter whose diameter is 10 µm or less is known as PM_10_, and a particulate matter whose diameter is 2·5 µm or less is known as PM_2·5_ or ultra-fine particulate [16]. In this study, carbon monoxide, nitrogen dioxide, ozone (O_3_), PM_10_, PM_2·5_, and sulfur trioxide (SO_2_) were used as variables.

The corresponding meteorological data were obtained from the National Climate Data Service System as weather variables. Both the automated synoptic observing data (of ASOS) provided by the “meteorological data open portal” of the Korea Meteorological Administration and the fine dust measuring data provided by Air Korea were combined and used based on region [17], date, and time. The weather data of Seoul City were used as reference data, and the maximum number of influence days of disease occurrence was assumed to be 3. Data on the average temperature, amount of precipitation, relative humidity, steam pressure, wind speed, and wind direction provided by the Korea Meteorological Administration were set as weather factors.

The distance between a regional emergency medical center in Seoul and an observatory was calculated. The five observatories with a small distance were selected. The mean of the values measured in the five observatories was calculated every hour. The mean of all the observatories in the region was also calculated. In this way, the mean value in the region was defined. A missing value was not processed and was left empty. The weather data from December 27, 2014, to December 31, 2017, were obtained. Seasons were classified as spring (March, April, and May); summer (June, July, and August); fall (September, October, and November); and winter (December, January, and February).

## 6. Result

### 6.1. Characteristics of Study Participants (Table 1)

A total of 18,619,252 patients visited EDs nationwide and 4,784,458 visited EDs in Seoul during the study period (Table 1). Among them, 525,579 patients were diagnosed with respiratory diseases (J code) according to the KCD. Respiratory disease patients accounted for 11.0% of the total ED patients. Among 525,579 patients who had visited EDs because of respiratory diseases within the 3-year period, 169,538 (32·3%) were reported in 2015, 202,114 (38·5%) in 2016, and 153,927 (29·3%) in 2017. The largest number of patients was reported in 2016. The average age was 28·1 ± 27·5 years. Specifically, 45% of these patients were aged 0–15 years, 37·6% were aged 16–60 years, and 17·4% were aged 61 years and older. Of the patients who visited EDs, the number of men (276,142, 52·5%) was higher than that of women. Approximately 52·2% of the patients visited EDs because of acute upper respiratory infections, which accounts for the highest number of patients in this subgroup. Pneumonia patients accounted for 15% of the total respiratory disease patients, and 43·8% of the hospitalized patients.

### 6.2. Analysis on the Number of Patients by Year, Month, Season, and Day (Table 2)

In the monthly analysis, of 525,579 patients who visited EDs because of respiratory diseases during the 3-year period, 71,122 (13·5%, the highest) occurred in December, 65,121 (12·4%, the second highest) in February, and 31,007 (5·95%, the lowest) in July (Table 2). In the seasonal analysis, 181,905 patients (34·6%, the highest) occurred in winter and 96,967 (18·4%, the lowest) in summer. In the days of the week analysis, 127,316 patients (13·9%, the highest) visited EDs on Sunday, while 60,077 (11·4%, the lowest) visited EDs on Thursday.

### 6.3. Characteristics of Weather Factors by Year (Table 3)

Of the weather factors, wind speed and wind direction showed a difference by year. Of the air pollution variables, nitrogen dioxide, O_3_, PM_2·5_, and SO_2_ showed a difference by year (Table 3).

### 6.4. Correlations between Weather Factors (Figure 1)

The correlations between six air pollution variables and six weather factors were analyzed, and whether multicollinearity existed was examined. Blue color indicated a negative correlation, while red color indicated a positive correlation. A darker color denoted more correlation between variables. Air pollution variables had positive correlations, while O_3_ had a negative correlation. Air pollution variables had negative correlations with weather factors (except for O_3_).

The correlation between six air pollution variables and six meteorological factors was compared. Blue color indicated a negative correlation, while red color indicated a positive correlation. A darker color denoted more correlation among the variables.

### 6.5. Results of Random Forest Based Analysis (Figures 2–6 and Table 4)

**Figure 2:**
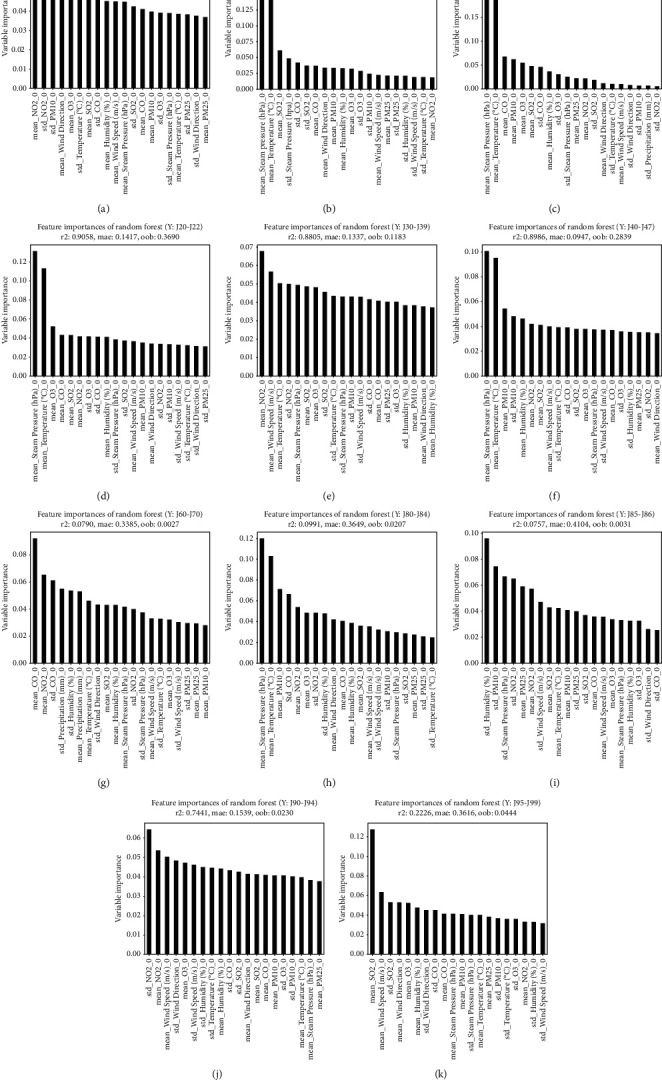
Analysis of the correlation between respiratory disease patients' visit to the ED and air pollution and weather variables (day 0). (a) [J00-J06] Acute upper respiratory infections. (b) [J09-J11] Influenza. (c) [J12-J18] Pneumonia. (d) [J20-J22] Other acute lower respiratory infections. (e) [J30-J39] Other diseases of upper respiratory tract. (f) [J40-J47] Chronic lower respiratory diseases. (g) [J60-J70] Lung diseases due to external agents. (h) [J80-J84] Other respiratory diseases principally affecting the interstitium. (i) [J85-J86] Suppurative and necrotic conditions of lower respiratory tract. (j) [J90-J94] Other diseases of pleura. (k) [J95-J99] Other diseases of the respiratory system.

**Figure 3:**
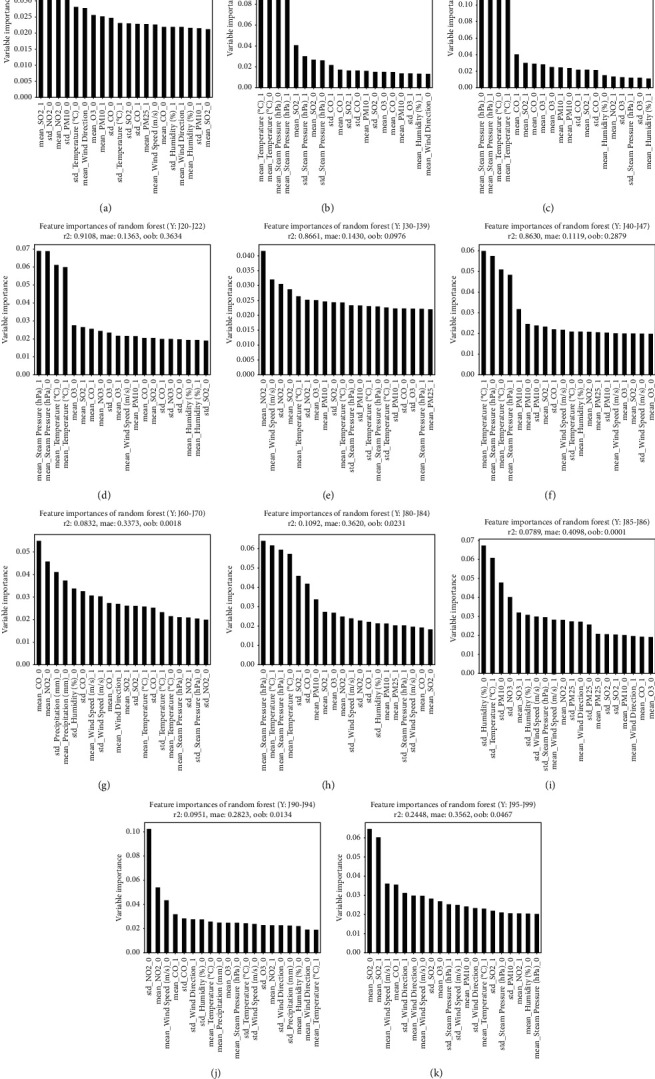
Analysis of the correlation between respiratory disease patients' visit to the ED and air pollution and weather variables (day 0–1). (a) [J00-J06] Acute upper respiratory infections. (b) [J09-J11] Influenza. (c) [J12-J18] Pneumonia. (d) [J20-J22] Other acute lower respiratory infections. (e) [J30-J39] Other diseases of upper respiratory tract. (f) [J40-J47] Chronic lower respiratory diseases. (g) [J60-J70] Lung diseases due to external agents. (h) [J80-J84] Other respiratory diseases principally affecting the interstitium. (i) [J85-J86] Suppurative and necrotic conditions of lower respiratory tract. (j) [J90-J94] Other diseases of pleura. (k) [J95-J99] Other diseases of the respiratory system.

**Figure 4:**
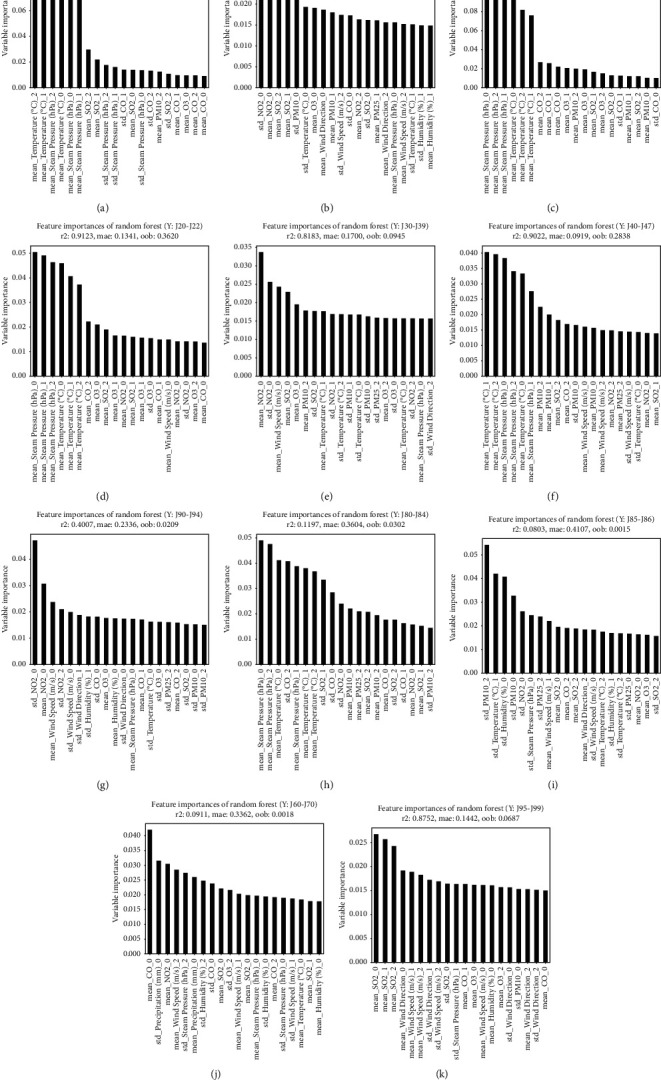
Analysis of the correlation between respiratory disease patients' visit to the ED and air pollution and weather variables (day 0–2). (a) [J00-J06] Acute upper respiratory infections. (b) [J09-J11] Influenza. (c) [J12-J18] Pneumonia. (d) [J20-J22] Other acute lower respiratory infections. (e) [J30-J39] Other diseases of upper respiratory tract. (f) [J40-J47] Chronic lower respiratory diseases. (g) [J60-J70] Lung diseases due to external agents. (h) [J80-J84] Other respiratory diseases principally affecting the interstitium. (i) [J85-J86] Suppurative and necrotic conditions of lower respiratory tract. (j) [J90-J94] Other diseases of pleura. (k) [J95-J99] Other diseases of the respiratory system.

**Figure 5:**
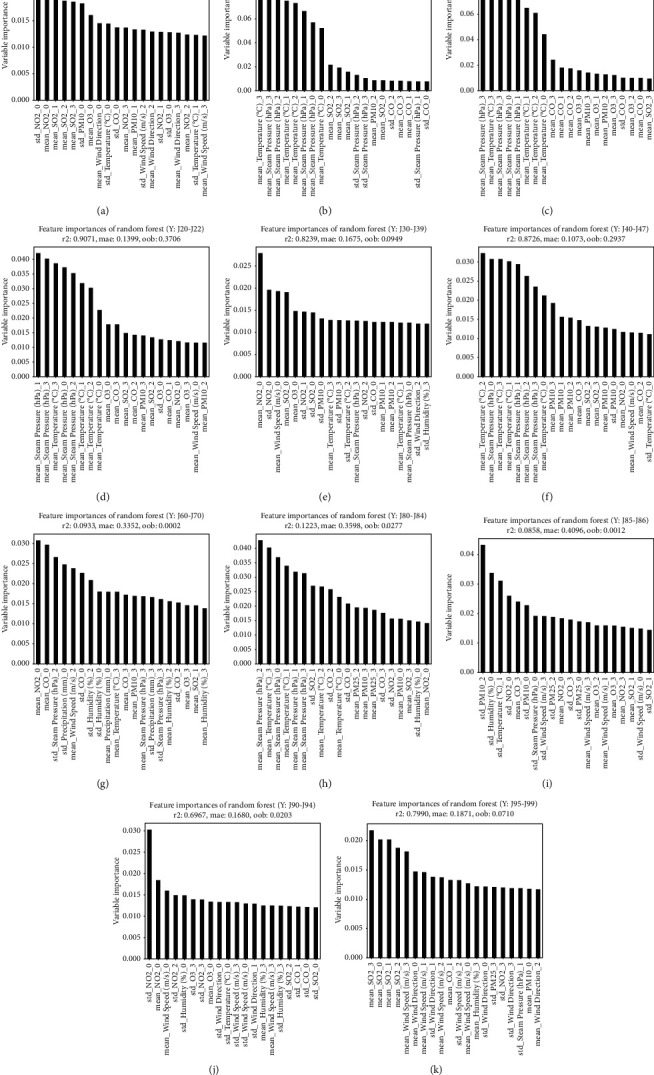
Analysis of the correlation between respiratory disease patients' visit to the ED and air pollution and weather variables (day 0–3). (a) [J00-J06] Acute upper respiratory infections. (b) [J09-J11] Influenza. (c) [J12-J18] Pneumonia. (d) [J20-J22] Other acute lower respiratory infections. (e) [J30-J39] Other diseases of upper respiratory tract. (f) [J40-J47] Chronic lower respiratory diseases. (g) [J60-J70] Lung diseases due to external agents. (h) [J80-J84] Other respiratory diseases principally affecting the interstitium. (i) [J85-J86] Suppurative and necrotic conditions of lower respiratory tract. (j) [J90-J94] Other diseases of pleura. (k) [J95-J99] Other diseases of the respiratory system.

**Figure 6:**
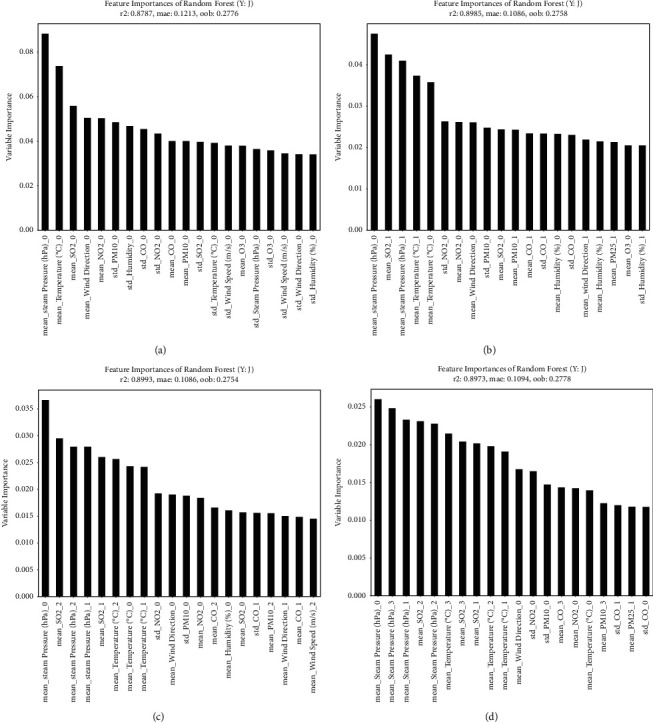
Analysis of the correlation between total respiratory disease patients' visit to the ED and air pollution and weather variables: (a) day 0, (b) days 0–1, (c) days 0–2, and (d) days 0–3.

Figures 2– 5 illustrate the graphs of 20 weather conditions and air pollution variables, which are highly related to the patients' visits to EDs because of each disease. Table 4 presents the top 10 variables. The number ranging from 0 to 3 after each variable denoted the relation between a patient's visit date and a variable measurement date. In other words, “0” indicates the relation between the weather condition on the day of a visit and an air pollution value; “1” indicates the relation between the weather condition on the day of a visit and the value on the day before the visit; “2” indicates the relation between the weather condition on the day of a visit and 2 days prior to the visit; and “3” indicates the relationship between the weather condition on the day of visit and 3 days prior to the visit. The “mean” is a value of the mean, while the “std” is a value of standard deviation that represents the changes in a variable on a certain day.  Figure 2 illustrates the weather and air pollution variables on the day of a visit that have high correlations with ED visit according to the patient's disease. Influenza, pneumonia, and other acute lower respiratory infections [J09–J11] were highly related to temperature and steam pressure (4B–D). Lung diseases due to external agents [J60–J70] were highly related to CO, NO_2_, and the amount of precipitation as air pollution variables (4G).  Figure 3 shows the correlations between the weather and air pollution variables on the day of a visit and the day before the visit and the ED visit.  Figure 4 presents the correlations between the variables on the day of a visit and 2 days before the visit.  Figure 5 illustrates the correlations between the weather and air pollution variables on the day of a visit, 2 days before the visit, and 3 days before the visit and the ED visit. A. Acute upper respiratory infection [J00–J06] was mainly related to NO_2_ on the day of a visit and to PM_10_ on the day of a visit and the day before the visit. B. Influenza was related to the temperature and steam pressure 3 days before a visit and was slightly influenced by PM_10_ 3 days before a visit. C. Pneumonia [J12–J18] was influenced by temperature and steam pressure 2–3 days before a visit, rather than on the day of the visit, and was influenced by PM_10_ as well.  Figure 6 is the result of total respiratory disease in this study. Steam pressure and SO_2_ are the most affective factors to visiting ED via respiratory diseases.

PM_10_ had high correlations with a patient's ED visit because of acute upper respiratory infections [J00–J06] and with days 0 and 1. In cases of influenza [J09–J11], pneumonia [J12–J18], other acute lower respiratory infections [J20–J22], and other diseases of the upper respiratory tract [J30–J39], day 0 was influential. In the case of chronic lower respiratory diseases [J40–J47], days 0, 1, 2, and 3 had high correlations with a patient's ED visit. In the case of suppurative and necrotic conditions of the lower respiratory tract [J85–J86], day 0 was influential (Table 4).

Among the climate factors, steam pressure had an effect on 0, 1, 2, 3 days, and among air pollution, NO_2_ had the most influence. Among the diseases that have the most frequent visits to the emergency department, the first acute upper respiratory infections [J00-J06] were affected by NO_2_, the second pneumonia [J12–J18] was affected by pressure, and the third influenza [J09–J11] was greatly affected by temperature. Regarding the second [J40-J47] chronic lower respiratory diseases, which is a disease that requires a lot of hospitalization, the temperature, and the fourth [J90-J94] other diseases of pleura, each NO2 value seems to be greatly affected by climate and pollutants (the first pneumonia [J12–J18], third [J00-J06] Acute upper respiratory infections mentioned above). PM10 affected the J85-86. With regard to PM_2·5_, in the case of other respiratory diseases principally affecting the interstitium [J80–J84], days 2 and 3 had high correlations with a patient's ED visit. In the case of suppurative and necrotic conditions of the lower respiratory tract [J85–J86], days 2 and 0 were influential (Table 4.)

## 7. Discussion

Based on the consistently registered and systemized data registry of national emergency medical centers, this study analyzed the correlations between weather and air pollution variables and respiratory disease patients visiting EDs by applying a machine learning approach as an AI technique. Previous studies have focused on the simple relationship between a single disease and one air factor. The present study considered all respiratory diseases and a variety of air pollution and weather variables. Unlike previous studies, it examined the effects of weather and air pollution variables 3 days before a visit. For air pollution, data of the five observatories in consideration of the location of the ED were used. Unlike previous studies that used the daily average data of air pollution variables [18, 19], this study utilized the data of 3 days before a visit, the daily temperature difference, and other data to determine the values of weather conditions in detail and identify their level of influence.

As a result, patients who visited EDs due to respiratory diseases had correlations with weather and air pollution variables on the day of the visit and 1–3 days before the visit. Of the air pollution variables, PM_10_ and PM_2·5,_ which have recently drawn a lot of attention, influenced patients' ED visit.

In this study, not only the effects of weather and air pollution variables on each disease, but also their level of influence was analyzed. Many air pollution variables had high correlations with acute upper respiratory infections [J00–J06], chronic lower respiratory diseases [J40–J47], and suppurative and necrotic conditions of the lower respiratory tract [J85–J86]. In cases of diseases that were highly influenced by air pollutants, steam pressure was not influential. As a result, steam pressure had a negative correlation with air pollution variables. In the case of acute upper respiratory infections [J00–J06], air pollution variables were highly influential; therefore, they had high correlations. Influenza and pneumonia were influenced by air factors like steam pressure; lower respiratory infections were influenced by air factors, and upper respiratory diseases by air pollution variables.

In the case of several diseases, compared with PM_2·5_, PM_10_ had a greater influence on patients' visit to ED. However, this does not mean that PM_2·5_ has little influence on the incidence of respiratory diseases. Nevertheless, it is reasonable to indicate that PM_10_ (larger particle size) is more influential on acute diseases that trigger a patient's visit to the ED during a short-term period (on the day of the visit to 3 days before the visit). More studies should be conducted to determine the long-term effects of PM_2·5_ [20], which is known to persist and affect the human body. PM10 influenced the respiratory disease patients' visits to the emergency departments.

In the case of influenza, the temperature and steam pressure on the day of a visit were most influential. In the case of pneumonia, which accounted for a majority of the respiratory disease patients visiting EDs, it was influenced more by steam pressure and temperature. The group of diseases including asthma (J40–J47) was influenced by PM_10_ following steam pressure. Acute upper respiratory infections were mostly influenced by air pollution variables, especially NO_2_ and PM_10_.

What was interesting was that acute upper respiratory infections [J00–J06], influenza [J09–J11], and pneumonia [J12–J18], which account for a majority of the respiratory diseases of patients visiting EDs, were highly influenced by PM_10_ following temperature and steam pressure and that PM_10_ was also highly influential in the top three diseases prompting visits to the ED: pneumonia [J12–J18], acute upper respiratory infections [J00–J06], and chronic lower respiratory diseases [J40–J47]. Therefore, of the air pollution variables, PM_10_ most influenced respiratory disease patients' visits to EDs.

Donaldson et al. reported that asthma symptoms were worsened by the influence of PM_10_. This finding is consistent with the results of the present study [21]. PM exposure can trigger an asthmatic response through multiple paths. Presumably, it is related to airway inflammation, increased smooth muscle constriction, direct stimulation of lipid mediators, additional oxidative stress, and proinflammatory burden [21, 22]. Other studies have also reported that an increase in PM_10_ is related to an increase in the use of asthma drugs [23, 24], According to a recent study conducted by Sohn et al. [25] in Korea, a daily temperature change influenced the pneumonia patients' visits to EDs in Seoul. Choi et al. [26] reported that maximum temperature, rainfall, relative humidity, and PM_10_ had correlations with community-acquired pneumonia. This study also revealed that pneumonia patients' visits to EDs were influenced by weather and air pollution variables, such as steam pressure, temperature, CO, PM_10_, and O_3_ (Figure 2(c)).

Arbex et al. (Brazil) [27] reported the correlations between acute upper respiratory infections [J00–J06] and air pollution variables. According to their report, the diseases were related to lag 0 of NO_2_, SO_2_, O_3_, and PM_10_. In this study, acute upper respiratory infections were also influenced by lag 0 in the order of NO_2_, *M*_10_, and SO_3_ (Figure 2(a)). Patients with acute upper respiratory infections accounted for 52.2% of the total respiratory disease patients visiting EDs and 12.8% of hospitalized patients. As such, the high number of patients with these diseases visiting the EDs was directly influenced by air pollution variables.

According to the research by Wanka et al. in Germany [28], weather and air pollution variables influenced respiratory diseases in a complex way. This study also revealed that a variety of variables were related to each other and influenced diverse disease groups in complex ways.

Zhang et al. [29] reported that a low concentration of PM_2·5_ was related to acute respiratory infections 3 days before a visit, while a high concentration of PM_2·5_ was related to the infections on the day before a visit. In this study, PM_2·5_ influenced acute respiratory infections in lag 0 and lag 2. Weather and air pollution variables were more directly influenced by respiratory diseases than other disease groups. A similar result was found for all the disease groups [30].

The number of respiratory disease patients will increase by day 3 when the values of steam pressure and temperature are low, and the values of air pollution variables are high. The weather-related health index for predicting respiratory disease patients visiting EDs is yet to be developed. If a prediction model is additionally developed based on the study results, it is possible to provide a fundamental material for preventing respiratory diseases related to weather changes and to help medical institutions utilize their facilities and manpower efficiently to manage patients with respiratory infections.

This study has the following limitations. First, the analysis was conducted with data that was already codified and collected; therefore, it was impossible to determine the clinical characteristics, prognosis, sources of infection, and underlying diseases of each patient. The primary outcome of this study was assessment of trends using large data. Therefore, it is necessary to analyze the clinical data of individual disease groups. Second, the study only lasted for 3 years. As described in this thesis, a group of chronic diseases and a group of acute diseases were included in the analysis. In particular, air pollution variables are needed in long-term influence analysis. However, the ED patients data system provided was based on 3-year data. Therefore, it is necessary to analyze the long-term influence of the study variables. Third, this study set the time lag to 3 days. If a general incubation period is taken into account, the lag of 14 days can be set. However, given the large number of variables, the time lag was set within a short-term period. At last, the data from the observatory near the hospital were used, not the data from the observatory near the patient's house. The reason for including the data from the observatory near the hospital is that if we use the observatory data near the patient's address, data cannot be obtained with personal information (address), and it has to be assumed that the patient has visited a nearby hospital.

In this study, the effects of weather and air pollution variables on respiratory disease patients' visits to EDs were analyzed. Most of the respiratory patients visiting EDs were diagnosed with acute upper respiratory infections [J00–J06], influenza [J09–J11], and pneumonia [J12–J18]. PM_10_ following temperature and steam pressure had influential relations with these diseases. In patients with pneumonia [J12–J18], acute upper respiratory infections [J00–J06], and chronic lower respiratory diseases [J40–J47] as the top three diseases managed in EDs, PM_10_ was highly influential. As a result, among air pollution variables, PM_10_ was found to influence the respiratory disease patients' visits to EDs. The number of respiratory disease patients visiting ED is expected to increase by day 3 when the values of steam pressure and temperature are low, and the variables of air pollution are high. Additionally, a respiratory disease prediction index must be established using a prediction model.

## Figures and Tables

**Table 1:** General characteristics.

	2015	2016	2017	Total
Total ED visits, n	1,535,931	1,632,922	1,615,605	··
Respiratory disease, *n* (%)	169,538 (32.3)	202,114 (38.5)	153,927 (29.3)	525,579
Age (mean ± SD)	27.1 (±27.1)	26.5 (±26.7)	31.2 (±28.7)	28.1 (±27.5)
0–15, *n* (%)	79,117 (46.7)	95,768 (47.4)	61,521(40.0)	236,406 (45.0)
16–60, *n* (%)	62,855 (37.1)	75,241 (37.2)	59,591 (38.7)	197,687 (37.6)
>60, *n* (%)	27,566 (16.3)	31,110 (15.4)	32,816 (21.3)	91,492 (17.4)
Male, *n* (%)	89,154 (52.6)	105,430 (52.2)	81,560 (53.0)	276,144 (52.5)

Diagnosis, n (%)				
[J00-J06] acute upper respiratory infections	93,307 (55.0)	102,199 (50.6)	78,789 (51.2)	274,295 (52.2)
[J09-J11] influenza	14,833 (8.7)	32,051(15.9)	13,617 (8.8)	60,501 (11.5)
[J12-18] pneumonia	26,150 (15.4)	28,759 (14.2)	23,863 (15.5)	78,772 (15.0)
[J20-J22] other acute lower respiratory infections	11,840 (7.0)	13,091 (6.5)	10,658 (6.9)	35,589 (6.8)
[J30-J39] other diseases of upper respiratory tract	3,120 (1.8)	3,565 (1.8)	4,122 (2.7)	10,807 (2.1)
[J40-J47] chronic lower respiratory diseases	11,028 (6.5)	12,393 (6.1)	11,491 (7.5)	34,912 (6.6)
[J60-J70] Lung diseases due to external agents	1,700 (1.0)	1,814 (0.9)	1,909 (1.2)	5,423 (1.0)
[J80-J84] other respiratory diseases principally affecting the interstitium	1,321 (0.8)	1,531 (0.8)	1,906 (1.2)	4,758 (0.9)
[J85-J86] suppurative and necrotic conditions of lower respiratory tract	221 (0.1)	290 (0.1)	278 (0.2)	789 (0.2)
[J90-J94] other diseases of pleura	4,809 (2.8)	4,869 (2.4)	5,041 (3.3)	14,719 (2.8)
[J95-J99] other diseases of the respiratory system	1,209 (0.7)	1,557 (0.8)	2,254 (1.5)	5,020 (1.0)
Admission, n (%)	34,354 (20.3)	38,789 (19.2)	33,971 (22.1)	107,123 (20.4)
[J00-J06] acute upper respiratory infections	4,643 (13.5)	5,254 (13.5)	3,833 (11.3)	13,730 (12.8)
[J09-J11] influenza	1,385 (4.0)	2,309 (6.0)	1,072 (3.2)	4,766 (4.4)
[J12-18] pneumonia	15,605 (45.4)	16,908 (43.6)	14,382 (42.3)	46,895 (43.8)
[J20-J22] other acute lower respiratory infections	2,018 (5.9)	2,443 (6.3)	2,036 (6.0)	6,497 (6.1)
[J30-J39] other diseases of upper respiratory tract	807 (2.3)	952 (2.5)	947 (2.8)	2,706 (2.5)
[J40-J47] chronic lower respiratory diseases	3,320 (9.7)	3,631 (9.4)	3,269 (9.6)	10,220 (9.5)
[J60-J70] Lung diseases due to external agents	1,130 (3.3)	1,215 (3.1)	1,267 (3.7)	3,612 (3.4)
[J80-J84] other respiratory diseases principally affecting the interstitium	968 (2.8)	1,180 (3.0)	1,516 (4.5)	3,664 (3.4)
[J85-J86] suppurative and necrotic conditions of lower respiratory tract	178 (0.5)	224 (0.6)	221 (0.7)	623 (0.6)
[J90-J94] other diseases of pleura	3,355 (9.8)	3,453 (8.9)	3,545 (10.4)	10,353 (9.7%)
[J95-J99] other diseases of the respiratory system	945 (2.8)	1,229 (3.2)	1,883 (5.5)	4,057 (3.8%)

**Table 2:** Distribution of respiratory disease incidence in ED by year, month, season, and days of week N (%).

	2015	2016	2017	Total
Monthly				
Jan	13,550 (8.0)	16,290 (8.1)	15,822 (10.3)	45,662 (8.7)
Feb	23,776 (14.0)	32,604 (16.1)	8,741 (5.7)	65,121 (12.4)
Mar	18,944 (11.2)	17,448 (8.6)	11,737 (7.6)	48,129 (9.2)
Apr	16,223 (9.6)	16,460 (8.1)	14,267 (9.3)	46,950 (8.9)
May	16,851 (9.9)	15,556 (7.7)	14,842 (9.6)	47,249 (9.0)
Jun	9,032 (5.3)	12,656 (6.3)	11,223 (7.3)	32,911 (6.3)
Jul	7,451 (4.4)	12,643 (6.3)	10,915 (7.1)	31,009 (5.9)
Aug	11,301 (6.7)	12,186 (6.0)	9,563 (6.2)	33,050 (6.3)
Sep	13,456 (7.9)	12,940 (6.4)	9,564 (6.2)	35,960 (6.8)
Oct	11,224 (6.6)	10,940 (5.4)	12,513 (8.1)	34,677 (6.6)
Nov	11,492 (6.8)	11,943 (5.9)	10,310 (6.7)	33,745 (6.4)
Dec	16,238 (9.6)	30,453 (15.1)	24,431 (15.9)	71,122 (13.5)

Seasonal				
Spring (3–5)	52,018 (30.7)	49,464 (24.5)	40,846 (26.5)	142,328 (27.1)
Summer (6–8)	27,784 (16.4)	37,485 (18.5)	31,701 (20.6)	96,970 (18.4)
Autumn (9–11)	36,172 (21.3)	35,823 (17.7)	32,387 (21.0)	104,382 (19.9)
Winter (12–2)	53,564 (31.6)	79,347 (39.3)	48,994 (31.8)	181,905 (34.6)

Days of week				
Sunday	40,462 (23.9)	49,179 (24.3)	37,675 (24.5)	127,316 (24.2)
Monday	22,688 (13.4)	28,708 (14.2)	21,591 (14.0)	72,987 (13.9)
Tuesday	19,421 (11.5)	24,952 (12.3)	18,126 (11.8)	62,499 (11.9)
Wednesday	19,418 (11.5)	22,903 (11.3)	18,143 (11.8)	60,464 (11.5)
Thursday	20,458 (12.1)	22,259 (11.0)	17,360 (11.3)	60,077 (11.4)
Friday	20,642 (12.2)	23,100 (11.4)	18,006 (11.7)	61,748 (11.7)
Saturday	26,449 (15.6)	31,018 (15.3)	23,027 (15.0)	80,494 (15.3)

**Table 3:** General information of atmospheric variables (Mean ± SD).

	2015		2016	2017	Total	P
Weather						
Precipitation (mm)		0.090 ± 0.293	0.113 ± 0.441	0.141 ± 0.641	0.115 ± 0.480	0.365
Temperature (°C)		13.614 ± 10.129	13.584 ± 11.023	13.054 ± 10.959	13.418 ± 10.705	0.729
Humidity (%)		59.785 ± 14.714	59.137 ± 13.967	57.744 ± 14.448	58.889 ± 14.391	0.147
Steam pressure (hPa)		11.115 ± 7.508	11.543 ± 8.111	11.058 ± 8.320	11.239 ± 7.983	0.668
Wind speed (m/s)		2.675 ± 0.901	2.276 ± 0.700	2.202 ± 0.684	2.384 ± 0.795	≤0.001
Wind direction (16 direction)		184.226 ± 68.367	199.434 ± 70.907	197.603 ± 64.954	193.760 ± 68.397	0.005

Air pollution						
CO	0.575 ± 0.186		0.570 ± 0.152	0.550 ± 0.165	0.565 ± 0.168	0.122
NO_2_	0.038 ± 0.012		0.038 ± 0.010	0.035 ± 0.011	0.037 ± 0.011	≤0.001
O_3_	0.020 ± 0.010		0.021 ± 0.010	0.022 ± 0.011	0.021 ± 0.010	0.024
PM10	47.547 ± 37.517		50.446 ± 21.878	47.267 ± 23.633	48.422 ± 28.551	0.249
PM2·5	23.007 ± 11.544		26.154 ± 11.496	24.652 ± 14.119	24.606 ± 12.501	0.003
SO2	0.006 ± 0.001		0.005 ± 0.001	0.005 ± 0.001	0.005 ± 0.001	≤0.001

CO, carbon monoxide; NO_2_, nitrogen dioxide; O_3_, ozone; PM, Particulate matter; SO2, sulfur dioxide.

**Table 4:** Analysis of the correlation between respiratory disease patients' ED visit and air pollution and weather variables.

KOICD	Time Lag	R2	Important Variables									
			1	2	3	4	5	6	7	8	9	10
J00-06	0	0·778	NO2 (mean, 0)	ste_NO2_0	PM10 (std, 0)	Wind direction (mean, 0)	SO2 (mean, 0)	Temperature (std, 0)	O3 (mean, 0)	CO (std, 0)	Humidity (mean, 0)	Steam pressure (mean, 0)
	0,1	0·8752	SO2 (mean, 1)	ste_NO2_0	NO2 (mean, 0)	PM10 (std, 0)	Wind direction (mean, 0)	Temperature (std, 0)	PM10 (std, 1)	CO (std, 0)	O3 (mean, 0)	Temperature (std, 1)
	0,1,2	0·8233	NO2 (std, 0)	SO2 (mean, 2)	NO2 (mean, 0)	SO2 (mean, 1)	PM10 (std, 0)	Wind direction (mean, 0)	Temperature (std, 0)	O3 (mean, 0)	Wind speed (std, 2)	
	0,1,2,3	0·882	NO2 (std, 0)	NO2 (mean, 0)	SO2 (mean, 1)	SO2 (mean, 3)	SO2 (mean, 2)	PM10 (std, 0)	O3 (mean, 0)	Wind direction (mean, 0)	Temperature (std, 0)	std_CO_ 0

J09-11	0	0·9504	Temperature (mean, 0)	Steam pressure (mean, 0)	SO2 (mean, 0)	Steam pressure (std, 0)	CO (std, 0)	CO (mean, 0)	PM10 (mean, 0)	SO2 (std, 0)	Humidity (mean, 0)	Wind direction (mean, 0)
	0,1	0·9529	Temperature (mean, 1)	Temperature (mean, 0)	Steam pressure (mean, 0)	Steam pressure (mean, 1)	SO2 (mean, 1)	Steam pressure (std, 0)	SO2 (mean, 0)	Steam pressure (std, 1)	CO (std, 1)	CO (mean, 1)
	0,1,2	0·9544	Temperature (mean, 1)	Temperature (mean, 2)	Temperature (mean, 0)	Steam pressure (mean, 2)	Steam pressure (mean, 0)	Steam pressure (mean, 1)	SO2 (mean, 2)	SO2 (mean, 1)	Steam pressure (std, 1)	Steam pressure (std, 2)
	0,1,2,3	0·882	NO2 (std, 0)	NO2 (mean, 0)	SO2 (mean, 1)	SO2 (mean, 3)	SO2 (mean, 2)	PM10 (std, 0)	O3 (mean, 0)	Wind direction (mean, 0)	Temperature (std, 0)	std_CO_ 0

J12-18	0	0·3447	Steam Pressure (mean, 0)	Temperature (mean, 0)	CO (mean, 0)	PM10 (mean, 0)	O3 (mean, 0)	SO2 (mean, 0)	CO (std, 0)	Humidity (mean, 0)	O3 (std, 0)	Steam Pressure (std, 0)
	0,1	0·3672	Steam pressure (mean, 0)	Steam pressure (mean, 1)	Temperature (mean, 0)	Temperature (mean, 1)	CO (mean, 1)	SO2 (mean, 1)	CO (mean, 0)	O3 (mean, 1)	O3 (mean, 0)	PM10 (mean, 1)
	0,1,2	0·3858	Steam pressure (mean, 0)	Steam pressure (mean, 2)	Steam pressure (mean, 1)	Temperature (mean, 0)	Temperature (mean, 2)	Temperature (mean, 1)	CO (mean, 2)	CO (mean, 1)	CO (mean, 0)	O3 (mean, 1)
	0,1,2,3	0·3941	Steam pressure (mean, 2)	Temperature (mean, 3)	Steam pressure (mean, 3)	Steam pressure (mean, 0)	Steam pressure (mean, 1)	Temperature (mean, 1)	Temperature (mean, 2)	Temperature (mean, 0)	CO (mean, 3)	CO (mean, 1)

J20-22	0	0·913	Steam pressure (mean, 0)	Temperature (mean, 0)	O3 (mean, 0)	SO2 (mean, 0)	NO2 (mean, 0)	O3 (std, 0)	Humidity (mean, 0)	CO (std, 0)	PM10 (mean, 0)	Steam pressure (std, 0)
	0,1	0·8325	Steam pressure (mean, 1)	Steam pressure (mean, 0)	Temperature (mean, 0)	Temperature (mean, 1)	O3 (mean, 0)	CO (mean, 1)	SO2 (mean, 1)	NO2 (mean, 0)	O3 (std, 0)	O3 (mean, 1)
	0,1,2	0·9067	Steam pressure (mean, 0)	Steam pressure (mean, 1)	Steam pressure (mean, 2)	Temperature (mean, 0)	Temperature (mean, 1)	Temperature (mean, 2)	O3 (mean, 0)	CO (mean, 2)	SO2 (mean, 2)	mean_PM10_
	0,1,2,3	0·898	Steam pressure (mean, 1)	Steam pressure (mean, 3)	Temperature (mean, 3)	Steam pressure (mean, 0)	Steam pressure (mean, 2)	O3 (mean, 0)	CO (mean, 3)	SO2 (mean, 3)	CO (mean, 2)	PM10 (mean, 3)

J30-39	0	0·814	NO2 (mean, 0)	Wind Speed (mean, 0)	Temperature (mean, 0)	Steam Pressure (mean, 0)	NO2 (std, 0)	SO2 (mean, 0)	O3 (mean, 0)	SO2 (std, 0)	Temperature (std, 0)	PM10 (std, 0)
	0,1	0·8772	NO2 (mean, 0)	Wind speed (mean, 0)	NO2 (std, 0)	SO2 (mean, 0)	Temperature (mean, 1)	O3 (mean, 0)	CO (mean, 1)	SO2 (mean, 1)	NO2 (mean, 0)	O3 (std, 0)
	0,1,2	0·8772	NO2 (mean, 0)	Wind speed (mean, 0)	NO2 (std, 0)	PM10 (mean, 2)	Temperature (mean, 1)	SO2 (std, 0)	O3 (mean, 0)	NO2 (std, 1)	Temperature (std, 2)	Temperature (std, 0)
	0,1,2,3	0·8213	NO2 (mean, 0)	SO2 (mean, 0)	NO2 (std, 0)	Wind speed (mean, 0)	O3 (mean, 0)	NO2 (std, 1)	SO2 (std, 0)	PM10 (std, 0)	Steam pressure (std, 0)	PM10 (std, 3)

J40-47	0	0·9008	Steam pressure (mean, 0)	Temperature (mean, 0)	PM10 (mean, 0)	PM10 (std, 0)	Humidity (mean, 0)	NO2 (mean, 0)	SO2 (mean, 0)	Wind speed (mean, 0)	Wind speed (std, 0)	CO (std, 0)
	0,1	0·8598	Temperature (mean, 1)	Steam pressure (mean, 0)	Steam pressure (mean, 1)	Temperature (mean, 0)	PM10 (mean, 1)	PM10 (mean, 0)	PM10 (std, 0)	SO2 (mean, 1)	Wind speed (mean, 0)	CO (std, 1)
	0,1,2	0·9005	Temperature (mean, 1)	Temperature (mean, 2)	Steam Pressure (mean, 0)	Temperature (mean, 0)	Steam Pressure (mean, 2)	Steam Pressure (mean, 1)	PM10 (mean, 2)	PM10 (mean, 1)	SO2 (mean, 2)	CO (mean, 2)
	0,1,2,3	0·87	Temperature (mean, 3)	Steam pressure (mean, 0)	Temperature (mean, 2)	Steam pressure (mean, 1)	Temperature (mean, 1)	Steam pressure (mean, 2)	Steam pressure (mean, 3)	Temperature (mean, 0)	PM10 (mean, 3)	PM10 (mean, 1)

J60-70	0	0·0789	CO (mean, 0)	NO2 (mean, 0)	CO (std, 0)	Precipitation (std, 0)	Humidity (std, 0)	Precipitation (mean, 0)	Temperature (mean, 0)	Humidity (mean, 0)	SO2 (mean, 0)	Wind direction (std, 0)
	0,1	0·0832	CO (mean, 0)	NO2 (mean, 0)	Precipitation (std, 0)	Precipitation (mean, 0)	Humidity (std, 0)	CO (std, 0)	Wind speed (std, 1)	CO (mean, 1)	Wind direction (mean, 1)	SO2 (mean, 1)
	0,1,2	0·0911	CO (mean, 0)	Precipitation (std, 0)	NO2 (mean, 0)	Wind speed (mean, 2)	Steam pressure (std, 2)	Precipitation (mean, 0)	Humidity (std, 2)	CO (std, 0)	SO2 (mean, 2)	O3 (std, 2)
	0,1,2,3	0·0933	NO2 (mean, 0)	CO (mean, 0)	Steam pressure (std, 2)	Precipitation (std, 0)	Wind speed (mean, 2)	std_CO_ 0	Humidity (std, 2)	Humidity (std, 0)	Precipitation (mean, 0)	Temperature (mean, 3)

J80-84	0	0·0991	Steam pressure (mean, 0)	Temperature (mean, 0)	PM10 (mean, 0)	std_C0_0	NO2 (mean, 0)	std__NO2_0	O3 (mean, 0)	Humidity (std, 0)	Wind direction (mean, 0)	CO (mean, 0)
	0,1	0·1092	Steam pressure (mean, 0)	Temperature (mean, 1)	Steam pressure (mean, 1)	Temperature (mean, 0)	SO2 (std, 1)	CO (std, 0)	PM10 (mean, 0)	SO2 (mean, 1)	O3 (mean, 0)	NO2 (mean, 0)
	0,1,2	0·1197	Steam Pressure (mean, 0)	Steam Pressure (mean, 2)	Temperature (mean, 0)	std_C0_2	Steam Pressure (mean, 1)	Temperature (mean, 1)	Temperature (mean, 2)	std__SO2_1	CO (std, 0)	NO2 (std, 2)
	0,1,2,3	0·1222	Steam pressure (mean, 2)	Temperature (mean, 0)	Steam pressure (mean, 0)	Temperature (mean, 1)	Steam pressure (mean, 1)	Steam pressure (mean, 3)	SO2 (std, 1)	Temperature (mean, 2)	CO (std, 2)	Temperature (mean, 0)

J85-86	0	0·0757	Humidity (std, 0)	PM10 (std, 0)	NO2 (std, 0)	Steam pressure (std, 0)	PM2·5 (mean, 0)	Wind speed (std, 0)	SO2 (mean, 0)	Temperature (mean, 0)	PM10 (mean, 0)	PM2·5 (std, 0)
	0,1	0·079	Humidity (std, 0)	Temperature (std, 1)	PM10 (std, 0)	NO2 (std, 0)	SO2 (mean, 1)	Humidity (std, 1)	Steam pressure (std, 0)	Wind speed (std, 0)	NO2 (mean, 0)	Wind speed (mean, 1)
	0,1,2	0·0803	PM10 (std, 2)	Temperature (std, 1)	Humidity (std, 0)	PM10 (std, 0)	NO2 (std, 0)	Steam pressure (std, 0)	PM2·5 (std, 2)	Wind speed (mean, 1)	SO2 (mean, 1)	CO (mean, 2)
	0,1,2,3	0·0858	PM10 (std, 2)	Humidity (std, 0)	Temperature (std, 1)	NO2 (std, 0)	mean_CO_ 3	PM10 (std, 0)	Steam pressure (std, 0)	Wind speed (std, 3)	PM2·5 (std, 2)	NO2 (mean, 0)

J90-94	0	0·0948	NO2 (std, 0)	NO2 (mean, 0)	Wind Speed (mean, 0)	Temperature (mean, 0)	CO (std, 0)	Wind Direction (std, 0)	Precipitation (std, 0)	O3 (mean, 0)	Steam Pressure (mean, 0)	O3 (std, 0)
	0,1	0·3804	NO2 (std, 0)	NO2 (mean, 0)	Wind speed (mean, 0)	Wind direction (std, 1)	Wind speed (std, 0)	Humidity (std, 0)	O3 (mean, 0)	Temperature (std, 0)	CO (mean, 1)	Wind direction (std, 0)
	0,1,2	0·3993	NO2 (std, 0)	NO2 (mean, 0)	Wind speed (mean, 0)	NO2 (std, 2)	Wind speed (std, 0)	CO (std, 0)	Wind direction (std, 1)	Humidity (std, 0)	Wind direction (std, 0)	Steam pressure (mean, 0)
	0,1,2,3	0·6921	NO2 (std, 0)	NO2 (mean, 0)	Wind speed (mean, 0)	NO2 (std, 2)	O3 (mean, 0)	O3 (std, 3)	NO2 (std, 3)	Wind direction (std, 1)	Wind direction (std, 0)	Wind speed (std, 0)

J95-99	0	0·1029	SO2 (mean, 0)	Wind speed (mean, 0)	SO2 (std, 0)	O3 (mean, 0)	Wind direction (mean, 0)	Wind direction (std, 0)	Humidity (mean, 0)	Steam pressure (std, 0)	PM10 (mean, 0)	CO (mean, 0)
	0,1	0·7786	SO2 (mean, 0)	SO2 (mean, 1)	Wind direction (std, 1)	Wind direction (mean, 0)	Wind speed (mean, 1)	Steam pressure (std, 1)	SO2 (std, 0)	Wind speed (mean, 0)	CO (mean, 1)	PM10 (std, 0)
	0,1,2	0·8723	SO2 (mean, 0)	SO2 (mean, 1)	SO2 (mean, 2)	Wind speed (mean, 2)	Wind speed (mean, 1)	Wind direction (mean, 0)	Wind direction (std, 1)	Wind speed (std, 2)	CO (mean, 1)	Steam pressure (std, 1)
	0,1,2,3	0·8743	SO2 (mean, 3)	SO2 (mean, 1)	SO2 (mean, 0)	SO2 (mean, 2)	Wind speed (mean, 3)	Wind direction (mean, 0)	Wind speed (mean, 1)	Wind speed (mean, 2)	Wind direction (std, 1)	PM2·5 (std, 3)

CO, carbon monoxide; NO_2_, nitrogen dioxide; O_3_, ozone; PM, Particulate matter; SO2, sulfur dioxide.

## Data Availability

Data sharing is not applicable to this article because the data that support the findings of this study are from NEDIS Korea. Restrictions apply to the availability of these data, which were used under license for this study.

## Appendix

Inclusion ICD-10 codes

- [J00-J06] Acute upper respiratory infections.
- [J09-J18] Influenza and pneumonia.
- [J20-J22] Other acute lower respiratory infections.
- [J30-J39] Other diseases of upper respiratory tract.
- [J40-J47] Chronic lower respiratory diseases.
- [J60-J70] Lung diseases due to external agents.
- [J80-J84] Other respiratory diseases principally affecting the interstitium.
- [J85-J86] Suppurative and necrotic conditions of lower respiratory tract.
- [J90-J94] Other diseases of pleura.
- [J95-J99] Other diseases of the respiratory system.
